# Antihyperglycemic effect of short-term arginyl-fructose supplementation in subjects with prediabetes and newly diagnosed type 2 diabetes: randomized, double-blinded, placebo-controlled trial

**DOI:** 10.1186/s13063-015-1036-z

**Published:** 2015-11-14

**Authors:** Su Eun Park, Ok-Hwan Kim, Jung Hyun Kwak, Kwang-Hyoung Lee, Young-In Kwon, Kwang Hoe Chung, Jong Ho Lee

**Affiliations:** Department of Food and Nutrition, National Research Laboratory for Clinical Nutrigenetics/Nutrigenomics, Yonsei University, Seoul, 120-749 Republic of Korea; Research Institute of Science for Aging, Yonsei University, Seoul, 120-749 Republic of Korea; R&D Center, CHA BioMed and Department of Applied Science, CHA University, Gyeonggi-do, 463-836 Republic of Korea; Department of Food and Nutrition, Hannam University, Daejeon, 305-811 Republic of Korea

**Keywords:** Glucose control, Prediabetes, Arginyl-fructose, Supplementation

## Abstract

**Background:**

A previous study reported that arginyl-fructose may have great value as a functional food with antioxidant and antidiabetic activities. However, there have been few clinical studies on the efficacy of arginyl-fructose supplementation for blood glucose control.

**Methods:**

In this double-blind, placebo-controlled study, 60 Korean subjects with prediabetes or type 2 diabetes mellitus were randomly assigned to placebo or test groups. The test group subjects received 1500 mg/day arginyl-fructose. Fasting serum levels of glucose, hemoglobin A1c, insulin, and free fatty acids were measured by 2-hour oral glucose tolerance tests at baseline and after the 6-week intervention. Eleven subjects dropped out or were excluded during the trial. The data for the remaining 49 were statistically analyzed using Student’s *t*-test and paired *t*-test.

**Results:**

After the 6-week intervention, the test group showed significant reductions in serum glucose levels at 30 minutes (−19.4 ± 5.62 mg/dL) and 60 minutes (−15.4 ± 7.01 mg/dL) and reduced glucose area under the curve (−27.4 ± 8.59 mg/dL) compared with those of the placebo control group. The changes (differences from baseline) in serum glucose levels at 60 minutes and glucose area under the curve in the test group differed significantly from those in the control group even after adjusting for baseline values. In contrast, glucose-related biomarkers including hemoglobin A1c, insulin, and C-peptide levels were not significantly improved by the dietary intervention with arginyl-fructose.

**Conclusions:**

Arginyl-fructose supplementation (1500 mg/day) may be beneficial for reducing postprandial blood glucose levels in patients with prediabetes or type 2 diabetes mellitus.

**Trial registration:**

ClinicalTrials.gov NCT02285231. Registered 11 May 2014.

**Electronic supplementary material:**

The online version of this article (doi:10.1186/s13063-015-1036-z) contains supplementary material, which is available to authorized users.

## Background

The prevalence of diabetes continues to increase globally. The number of people with diabetes is predicted to increase to 366 million in 2030 [1, 2]. The 2012 Korean National Health and Nutrition Survey indicates that the prevalence of diabetes mellitus and impaired fasting glucose (IFG) were 11.8 and 22.2 %, respectively, in Korean adults (≥30 years old). In Korea, the number of people with IFG and impaired glucose tolerance (IGT), which are prediabetic conditions, is increasing every year [3].

Many foods and supplements have been reported to promote normal glucose control. Korean red ginseng (KRG; *Panax ginseng* C.A. Meyer) is a well-known traditional oriental medicine and tonic [4]. Red ginseng is processed for use by steaming and drying raw ginseng. One of the processing steps involves a nonenzymatic browning reaction called a Maillard reaction. Arginyl-fructose (AF) and arginyl-fructosyl-glucose (AFG) are major Amadori rearrangement compounds formed during the early stage of the Maillard reaction from arginine and glucose or arginine and maltose, respectively [5].

Arginine is a primary amino acid constituent of ginseng. Amino acid derivatives such as AF and AFG are widely distributed in KRG [5–7]. Previous studies reported that commercial red ginseng products could have AF and AFG levels ranging from 0.35 − 2.51 % and 0.25 − 2.64 %, respectively [8, 9]. The ginsenosides, which are better-known KRG components, have a content ranging from 0.5 − 1.1 % [8, 10].

Many studies have evaluated the pharmacological effects of AF and AFG. One study reported that AF and AFG have antihyperglycemic effects in vitro and in vivo. These Amadori rearrangement compounds (AF and AFG) significantly reduced postprandial blood glucose levels after starch or sucrose loading in rats. AF and AFG displayed mild inhibitory activity against pancreatic α-amylase [8] and moderate levels of cellular antioxidant activity [11]. Although AF and AFG have potential as pharmacological agents for glycemic control, there are no clinical trials assessing the effects of AF and AFG on blood glucose regulation.

Our study is the first evaluation of AF supplementation for glycemic control in humans. Our previous study indicated that KRG supplementation was efficacious for glucose control [12]. Therefore, we applied a similar study design for this randomized, double-blind, and placebo-controlled clinical trial to evaluate the effect of AF supplementation on blood glucose control in subjects with IFG, IGT, or newly diagnosed type 2 diabetes mellitus (T2DM). This study establishes clinical evidence for the effect of AF on blood glucose control.

## Methods

### Subjects and study design

Study participants aged 20–70 years old were recruited by advertisements in a local newspaper. All subjects were evaluated as prediabetic (IFG or IGT) or were newly diagnosed with T2DM and not taking any hyperglycemic medicine. We measured fasting glucose levels from oral glucose tolerance tests (OGTTs; 75 g oral glucose) at 2-hour time points, and confirmed diagnoses of T2DM, IFG, or IGT. Prediabetes was defined as having a serum glucose level 100–125 mg/dL (IFG) or 2-hour OGTT value 140–199 mg/dL (IGT). The T2DM diagnosis was defined as fasting plasma glucose ≥126 mg/dL and 2-hour OGTT value ≥200 mg/dL. Subjects were excluded if they had any diagnosis of renal disease, liver disease, chronic inflammatory disease, chronic alcoholism, consumed any health-related functional food, were pregnant, were breast-feeding, or were taking glucose-lowering medications or insulin injections. After the glucose screening test and evaluation of exclusion criteria, subjects with IFG, IGT, or newly diagnosed T2DM were enrolled in this study. Sixty subjects (20–70 years old) with IFG, IGT, or T2DM gave written informed consent for enrollment in this study, which was approved by the Institutional Review Board of Yonsei University. The study is registered at ClinicalTrials.gov (trial number NCT02285231).

AF and placebo were both provided by CHA BioMed Co., Ltd (Gyeonggi-do, Korea). AF was manufactured by heat treatment of food-grade arginine and glucose, which were mixed, stirred for 1 hour at 80 °C, and subjected to spray drying. Participants in the test group received one AF tablet per day (1500 mg/day). The placebo group received one tablet containing baked barley powder per day (1500 mg/day). This study was designed as a 6-week randomized, double-blinded, placebo-controlled trial. Sixty subjects were randomly assigned to receive placebo (*n* = 30) or AF (*n* = 30). The participants were randomized in a 1:1 ratio for the intervention or the placebo group. Both groups consumed two capsules, three times daily, for a total of six capsules consumed per day. Subjects met with the investigational team at three different time points: during screening (week −2), during randomization and treatment baseline (week 0), and at the treatment end-point (week 6). All participants were encouraged to maintain their usual lifestyle and dietary habits and to keep a food record. Compliance was assessed by counting the remaining tablets at week 6 and by evaluating the food records. If more than 80 % of the tablets were consumed, compliance was considered to be acceptable.

### Sample size calculation

Previous research on the effects of glucose-related functional foods on patients with IFG, IGT, or T2DM reported a 19.24 ± 2.86 mg/dL (± standard deviation (SD)) reduction in OGTT glucose levels at 120 minutes compared with placebo effects. We hypothesized that a similar reduction in OGTT glucose levels at 120 minutes would be observed after AF treatment among the target population in this study, which is considered clinically meaningful [13]. Power analysis indicated that a total sample of 40 subjects, 20 in each group, would be required to detect a 19.24 mg/dL reduction in glucose at 120 minutes, with a significance level of 0.05 and at least 80 % power. We factored in a 30 % dropout rate to determine a recruitment target of 58 study subjects.

### Dietary intervention and assessment of dietary intake/physical activity level

The diet information for the subjects was obtained using both a 24-hour recall method and a semiquantitative food frequency questionnaire that had been previously validated [14]. We used the former for analyses and the latter to check if the 24-hour recall method yielded representative data for the usual dietary pattern. All subjects were given written and verbal instructions by a dietitian on how to complete 3-day (2 weekdays and 1 weekend day) dietary records every week throughout the study period. For the dietary records, subjects were instructed to weigh and record the food amounts before ingestion and any food remaining after ingestion. The dietician checked participants’ compliance via biweekly telephone interviews during the intervention period. Participants were interviewed to discern whether they were complying with the study instructions, including AF supplement consumption, dietary intake, and weight change. Dietary energy values and nutrient contents from the 3-day food records were calculated using the Computer Aided Nutritional analysis program (CAN-pro 3.0, Korean Nutrition Society, Seoul, Korea). Total energy expenditure (kcal/day) was calculated from activity patterns including basal metabolic rate, physical activity for 24 hours [15], and specific food dynamic action. The basal metabolic rate for each subject was calculated with the Harris-Benedict equation [16].

### Anthropometric parameters, blood pressure measurements, and blood collection

Body weight and height were measured in the morning with the subjects unclothed and without shoes. Body mass index (BMI) was calculated as body weight in kg divided by height in meters squared (kg/m^2^). Body composition was determined by a foot-to-foot bioelectrical impedance analyzer while the subject was standing erect with bare feet on the analyzer footpads (Tanita, Japan); the analyzer was used to obtain lean body mass (kg), fat mass (kg), and body fat (%). Blood pressure was measured after a 10-minute rest using the left arm of the seated patient with an automatic blood pressure monitor (TM-2654, A&D, Tokyo, Japan). The mean of two measurements was recorded for each subject. Venous blood specimens were collected in EDTA-treated and plain tubes after a 12-hour fast. The tubes were placed on ice until they arrived at the laboratory (within 1–3 hours), where they were centrifuged into plasma or serum and then stored at −70 °C until analysis.

### Glucose, insulin, and C-peptide concentrations

Fasting glucose was determined using the glucose oxidase method with a Beckman Glucose Analyzer (Beckman Instruments, Irvine, CA, USA). Insulin was determined by radio-immunoassay using commercial kits from Immuno-Nuclear Corporation (Stillwater, MN, USA). C-peptide concentration was determined by two-site sandwich immunoassay using an ADVIA Centaur XP Immunoassay system (Siemens, USA).

### Oral glucose tolerance test

Subjects drank a 75-g glucose solution after an overnight fast. Venous blood specimens were collected before and 30, 60, and 120 minutes after glucose ingestion. Glucose was determined using the glucose oxidase method with the Beckman Glucose Analyzer (Beckman Instruments).

### Serum lipid profile and apolipoprotein A-I and B

Fasting serum concentrations of total cholesterol and triglyceride (TG) were measured using commercially available kits and the Hitachi 7150 Autoanalyzer (Hitachi Ltd., Tokyo, Japan). High-density lipoprotein (HDL) was measured from the supernatant using enzymatic methods. Low-density lipoprotein (LDL) was estimated indirectly for subjects with serum TG levels <400 mg/dL using the Friedewald formula. For subjects with serum TG concentrations ≥400 mg/dL, LDL was determined directly using an enzymatic method and the Hitachi 7150 Autoanalyzer. Serum apolipoprotein (apo) A-I and apo B were determined using turbidimetry at 340 nm using a specific anti-serum (Roche, Switzerland).

### Statistical analyses

Statistical analyses were computed with SPSS v. 18.0 for Windows (SPSS Inc., Chicago, IL, USA). Each variable was examined for normal distribution, and significantly skewed variables were subjected to log transformation. For descriptive purposes, mean values of untransformed and unadjusted variables are presented. Baseline characteristics and comparisons between the intervention and control groups were evaluated using the Student’s *t*-test for continuous variables. A paired *t*-test was used to evaluate the effects of AF or placebo within each group before and after the intervention. Only those results from participants who completed the intervention program were analyzed (*n* = 49). Results are expressed as mean ± standard error of the mean (SEM). A *p*-value <0.05 was considered statistically significant.

## Results

### Characteristics of study participants

**Fig. 1:**
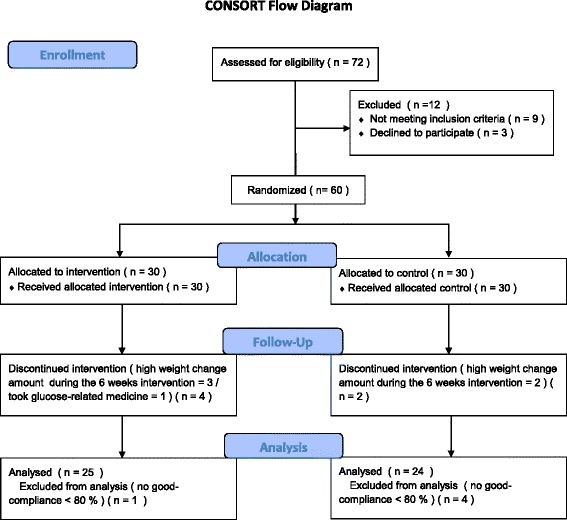
Flow-chart of study randomization, allocation, follow-up, and analysis

Among the enrolled subjects (*n* = 60), 11 subjects dropped out and 49 subjects completed the study. Study participant were included in the trial from January 2014 to May 2014. One of the 11 dropout subjects was in the test group and was excluded due to the consumption of glucose-related medicine during the trial. Three subjects in the test group and two subjects in the placebo group dropped out due to large weight changes (more than ±2 kg) during the trial. Five subjects (one from the test group and four from the placebo group) were excluded for suboptimal compliance with ingesting AF (consuming <80 % of the instructed amount). Finally, 49 participants (test group, 25; placebo group, 24) completed the study. Sex distribution and age did not significantly differ between the test and placebo groups (Fig. 1). No adverse events or side effects were seen in any participants. For the Consort checklist, see Additional file 1.

General baseline characteristics for the 49 subjects were evaluated (Table 1). There were no significant differences between the two groups with respect to baseline values for age, BMI, waist diameter, waist-to-hip ratio, P-fat, lean body mass, and blood pressure (systolic and diastolic). When the subjects’ parameters were compared before and after treatment, there were significant reductions in weight, BMI, waist diameter, and P-fat in the placebo group after 6 weeks. In the test group, weight and BMI significantly declined after the intervention compared with baseline values. However, these changes were not significantly different between the two groups. Total calorie intake (TCI), total energy expenditure (TEE), and dietary macronutrient intake did not significantly differ between the two groups at baseline. After the 6-week intervention, the placebo group showed significant increases in TEE and decreases in TCI compared with baseline. TCI was significantly reduced compared with baseline in the test group after the 6-week intervention. However, the net changes in these variables were not significantly different between the two groups (data not shown).

**Table 1:** Characteristics of study participants

	Placebo (*n* = 24)	Test (*n* = 25)
Age (years)	58.3 ± 1.27	56.6 ± 1.98
Sex (male/female)	(19/5)	(19/6)
Weight (kg)		
0 week	65.9 ± 2.25	70.3 ± 1.92
6 week	65.3 ± 2.25^**^	69.67 ± 1.86^**^
BMI (kg/m^2^)		
0 week	23.4 ± 0.67	24.9 ± 0.57
6 week	23.2 ± 0.67^**^	24.7 ± 0.55^**^
WHR (cm/cm)		
0 week	0.89 ± 0.01	0.92 ± 0.01
6 week	0.89 ± 0.01	0.92 ± 0.01
Body fat (%)		
0 week	23.4 ± 1.44	26.1 ± 1.45
6 week	22.7 ± 1.49^**^	26.1 ± 1.48
Lean body mass (kg)		
0 week	50.2 ± 1.55	51.9 ± 2.94
6 week	50.1 ± 1.55	51.3 ± 1.57
Systolic BP		
0 week	128.6 ± 2.67	129.5 ± 2.94
6 week	124.5 ± 2.99^†^	128.2 ± 2.17
Diastolic BP		
0 week	77.9 ± 1.55	78.2 ± 1.85
6 week	76.4 ± 1.70	78.2 ± 1.57
TEE (kcal/day)		
0 week	2123 ± 232.52	2266 ± 273.43
6 week	2140 ± 229.63^*^	2280 ± 280.27
TCI (kcal/day)		
0 week	2241 ± 251.81	2288 ± 261.65
6 week	2210 ± 261.45^*^	2266 ± 252.84^*^
Carbohydrate (%)		
0 week	61.9 ± 0.64	61.3 ± 0.81
6 week	61.8 ± 0.79	61.3 ± 0.77
Protein (%)		
0 week	15.9 ± 0.38	16.0 ± 0.37
6 week	15.8 ± 0.36	15.9 ± 0.38
Fat (%)		
0 week	22.9 ± 1.10	23.4 ± 1.25
6 week	23.1 ± 1.12	23.4 ± 0.87

Data are shown as mean ± SEM

^†^ *p* < 0.1, *p < 0.05, **p < 0.01, compared to 0 week, tested by paired *t*-test

*BMI* Body mass index, *BP* Blood pressure, *TCI* Total calorie intake, *TEE* Total energy expenditure, *WHR* Waist-to-hip ratio

### Effect of arginyl-fructose supplementation on postprandial glucose metabolism

**Fig. 2:**
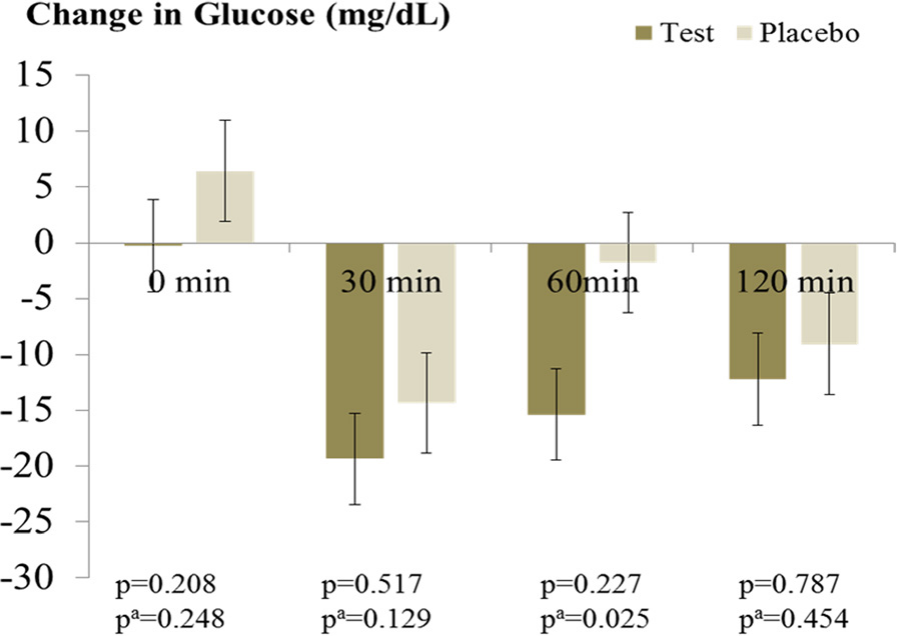
Effect of a 6-week trial of arginyl-fructose supplementation on serum blood glucose levels at 2 hours after glucose ingestion, determined using oral glucose tolerance test, in subjects with prediabetes and newly diagnosed type 2 diabetes. *p*-values derived from independent Student’s *t*-test, ^a^ *p*-values derived after adjustment for each baseline

**Fig. 3:**
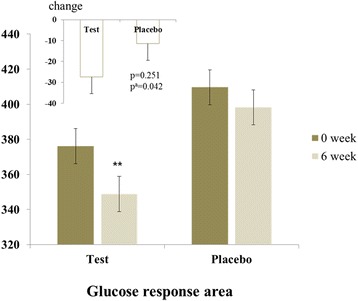
Effect of a 6-week trial of arginyl-fructose supplementation on glucose response area of subjects with prediabetes and newly diagnosed type 2 diabetes, determined using oral glucose tolerance tests. *p*-values derived from independent Student’s *t*-test, ^a^ *p*-values derived after adjusting for each baseline. **p < 0.01, tested by paired *t*-test

Serum glucose levels at baseline and after the 6-week intervention in test and placebo groups are presented in Table 2. After the intervention, the test group showed significant decreases in OGTT serum glucose levels at 30 minutes (−19.4 ± 5.62, *p* = 0.002) and 60 minutes (−15.4 ± 7.01, *p* = 0.035) and glucose area under the curve (AUC) (−27.4 ± 8.59, *p* = 0.002). After the 6-week intervention, the placebo group showed a significant decrease in OGTT serum glucose levels at 30 minutes (−14.3 ± 5.24, *p* = 0.015). After adjustment for baseline values, net changes in serum glucose levels at 60 minutes and glucose AUC in the test group were significantly larger than those in the placebo group after 6 weeks of AF intervention (Figs. 2 and 3).

**Table 2:** Oral glucose tolerance test serum glucose levels at baseline and after the 6-week intervention

	Placebo (*n* = 24)	Test (*n* = 25)
Glucose (mg/dL)^a^		
0 minutes		
0 week	114.0 ± 2.50	114.6 ± 2.19
6 week	120.4 ± 3.54	114.4 ± 4.35
30 minutes		
0 week	212.6 ± 7.78	197.9 ± 8.11
6 week	198.3 ± 6.42^*^	178.6 ± 7.36^**^
60 minutes		
0 week	235.4 ± 10.61	209.3 ± 11.70
6 week	233.6 ± 6.79	194.0 ± 11.59^*^
120 minutes		
0 week	196.6 ± 12.01	183.1 ± 13.19
6 week	187.5 ± 8.68	170.9 ± 13.39
Glucose AUC (mg/dL per hour)^a^		
0 week	409.7 ± 15.61	376.2 ± 17.38
6 week	398.2 ± 11.14	348.8 ± 16.55^**^

Data are shown as mean ± SEM

^†^ *p* < 0.1, *p < 0.05, **p < 0.01, compared to 0 week, tested by paired *t*-test

^a^Analyzed after log transformation

*AUC* Area under the curve

### Levels of glucose-related biomarkers

Glycosylated hemoglobin A1c (HbA1c) levels were significantly lower in test and control groups after the 6-week intervention compared with baseline values (*p* = 0.000 and *p* = 0.007, respectively). However, this change was not significantly different between the two groups. After the intervention, significant changes were observed in the test group serum insulin levels at 60 minutes (*p* = 0.010) and insulin AUC (*p* = 0.017) compared with baseline levels (Table 3). In the placebo group, serum insulin levels at 30 minutes (*p* = 0.012) and 60 minutes (*p* = 0.032) and insulin AUC (*p* = 0.003) significantly decreased compared with baseline after the intervention. There were also significant decreases in serum C-peptide levels at 30 minutes (*p* = 0.018) and 60 minutes (*p* = 0.025) and C-peptide AUC value (*p* = 0.044) in the placebo group. However, the net changes in these variables were not significantly different between the two groups.

**Table 3:** Glucose-related biomarkers at baseline and after the 6-week intervention

	Placebo (*n* = 24)	Test (*n* = 25)
Hemoglobin A1c (%)^a^		
0 week	6.4 ± 0.16	6.4 ± 0.14
6 week	6.2 ± 0.14^***^	6.1 ± 0.13^**^
Insulin (uU/mL)^a^		
0 minutes		
0 week	9.5 ± 0.77	11.1 ± 1.03
6 week	9.2 ± 0.68	12.4 ± 2.49
30 minutes		
0 week	30.5 ± 3.93	43.7 ± 8.26
6 week	21.3 ± 3.03^*^	41.3 ± 8.60
60 minutes		
0 week	44.5 ± 6.35	58.5 ± 9.34
6 week	34.9 ± 5.06^*^	46.3 ± 8.21^*^
120 minutes		
0 week	46.7 ± 7.65	56.1 ± 10.58
6 week	36.3 ± 6.49^†^	42.1 ± 7.46^†^
Insulin AUC (uU/mL per hour)^a^		
0 week	74.3 ± 8.66	96.6 ± 13.98
6 week	57.3 ± 7.72^**^	79.5 ± 11.74^*^
C-peptide (ng/mL)^a^		
0 minutes		
0 week	2.1 ± 0.16	2.5 ± 0.18
6 week	2.1 ± 0.15	2.7 ± 0.33
30 minutes		
0 week	5.1 ± 0.38	6.0 ± 0.60
6 week	4.4 ± 0.39^*^	5.9 ± 0.71
60 minutes		
0 week	7.5 ± 0.49	8.6 ± 0.75
6 week	6.8 ± 0.47^*^	8.0 ± 0.68
120 minutes		
0 week	9.4 ± 0.67	9.1 ± 0.69
6 week	9.2 ± 0.65	8.4 ± 0.64
C-peptide AUC (ng/mL)^a^		
0 week	13.4 ± 0.79	14.6 ± 1.10
6 week	12.4 ± 0.83^*^	13.8 ± 1.01

Data are shown as mean ± SEM

^†^ *p* < 0.1, *p < 0.05, **p < 0.01, ***p < 0.001 compared to 0 week, tested by paired *t*-test

^a^Analyzed after log transformation

*AUC* Area under the curve

### Lipid profiles and laboratory measurements

No significant differences in the test and placebo groups were observed for serum lipid profiles. After the intervention, the serum albumin level significantly decreased in both groups. There were no significant differences in white blood cell counts, creatinine, blood urea nitrogen, serum glutamic oxaloacetic transaminase, serum glutamate-pyruvate transaminase, or high-sensititvity C reactive peptide within each group before and after the intervention (data not shown).

## Discussion

This study determined the effects of 6 weeks AF supplementation on glucose control in 49 Korean subjects with prediabetes and newly diagnosed T2DM. After the intervention, the test group showed significant decreases in OGTT serum glucose levels at 30 and 60 minutes and glucose AUC. After adjusting for baseline levels, the changes (differences from baseline) in OGTT serum glucose levels at 60 minutes and glucose response areas in the AF intervention group were significantly different from those in the placebo group. These results indicate that AF supplementation for 6 weeks may have beneficial effects for reducing postprandial blood glucose levels in individuals with IFG, IGT, or newly diagnosed T2DM. The AF content in KRG is relatively high. This compound is formed from L-arginine plus glucose or maltose via a Maillard reaction that occurs during commercial processing of raw ginseng to produce the dried red ginseng product [5].

Previous studies reported that AF and AFG have strong and similar sucrase inhibitor activity. Ha et al. reported that AF had an antihyperglycemic effect and inhibited postprandial hyperglycemia in vitro and in vivo [8]. They demonstrated that AF has strong sucrase and α-amylase inhibitory activity in vitro. The carbohydrate hydrolyzing enzymes α-amylase and α-glucosidase regulate glucose digestion and absorption. Therefore, a therapeutic approach to inhibit postprandial hyperglycemia is to inhibit the activity of these enzymes using α-glucosidase inhibitors such as acarbose [17, 18]. In an animal model, AF significantly reduced postprandial blood glucose levels after starch or sucrose loading in Sprague–Dawley rats. These results indicate that AF may exert antidiabetic effects by suppressing carbohydrate absorption in the gastrointestinal tract, and thereby reduce the postprandial increase in blood glucose levels [8]. The latest study from the same authors (2014) evaluated whether AF could lower blood glucose levels in db/db mice (T2DM model), and the results and conclusions were similar [19]. The administration of AF for 42 days with high carbohydrate diets decreased fasting blood glucose levels, HbA1c concentrations, and total body weight. These reductions were similar to those observed after acarbose treatment, and were significantly lower than controls (*p* < 0.001). These results indicate that AF effectively manages hyperglycemia. Therefore, this trial provides evidence in support of potential therapeutic applications of AF to control T2DM.

The antioxidant activities of AF and AFG were investigated previously; these compounds exhibited strong radical scavenging activities. AF has greater radical scavenging activity for hydroxyl radical and DPPH than AFG [20, 21]. This result was similar to a previous study, which proposed that the Maillard reaction products AF and AFG could be valuable materials for the development of nutraceutical food with antioxidant activity [11].

Our study is the first clinical trial of AF supplementation for controlling postprandial hyperglycemia in prediabetic and newly diagnosed T2DM subjects. The randomized controlled trial enrolled Korean adult subjects, and determined that AF supplementation may reduce postprandial glucose levels. Our study has some limitations. The trial had a relatively short 6-week duration and enrolled a small number of participants. In addition, the screening criteria for determining if subjects were prediabetic or newly diagnosed T2DM were quite broad. Therefore, further study is needed using a larger number of participants for a longer time with subjects who have IGT or IFG alone. For future large-scale studies, additional testing is needed to optimize AF supplementation dosages and glucose control.

## Conclusions

Our study determined the effects of 6 weeks AF supplementation on glucose control in 49 Korean subjects with prediabetes and newly diagnosed T2DM. After the 6-week intervention, the test group showed significant reductions in serum glucose levels at 30 minutes (−19.4 ± 5.62 mg/dL) and 60 minutes (−15.4 ± 7.01 mg/dL) and reduced glucose AUC (−27.4 ± 8.59 mg/dL) compared with those of the placebo control group. The changes (differences from baseline) in serum glucose levels at 60 minutes and glucose AUC in the test group differed significantly from those in the control group even after adjusting for baseline values. In conclusion, AF supplementation (1500 mg/day) may be beneficial for reducing postprandial blood glucose levels in patients with prediabetes or T2DM.

## Additional file

Additional file 1:
**CONSORT Checklist.** (DOC 60 kb)

## Abbreviations

AF: Arginyl-fructose
AFG: Arginyl-fructose-glucose
apo: Apolipoprotein
AUV: Area under the curve
BMI: Body mass index
HbA1c: Hemoglobin A1c
HDL: High-density lipoprotein
IFG: Impaired fasting glucose
IGT: Impaired glucose tolerance
KRG: Korean red ginseng
LDL: Low-density lipoprotein
OGTT: Oral glucose tolerance test
T2DM: Type 2 diabetes mellitus
TCI: Total calorie intake
TEE: Total energy expenditure
TG: Triglyceride
